# Effect of a Sulfur Precursor on the Hydrothermal Synthesis of Cu_2_MnSnS_4_

**DOI:** 10.3390/ma14133457

**Published:** 2021-06-22

**Authors:** Edyta Waluś, Maciej Manecki, Grzegorz Cios, Tomasz Tokarski

**Affiliations:** 1 Department of Mineralogy, Petrography, and Geochemistry, AGH University of Science and Technology, al. Mickiewicza 30, 30-059 Kraków, Poland; gpmmanec@cyf-kr.edu.pl; 2 Department of Earth Sciences, Uppsala University, Villavägen 16, SE-752 36 Uppsala, Sweden; 3 Academic Centre for Materials and Nanotechnology, AGH University of Science and Technology, al. Mickiewicza 30, 30-059 Kraków, Poland; ciosu@agh.edu.pl (G.C.); tokarski@agh.edu.pl (T.T.)

**Keywords:** CMTS, hexagonal, tetragonal, microspheres, thiourea, Na_2_S

## Abstract

Cu_2_MnSnS_4_ (CMTS) is acknowledged as an alternative to traditional semiconductors. The structure and microstructure of synthetic CMTS depend on, among other things, the types of sulfur sources used. Traditionally obtained CMTS mostly has a tetragonal structure. In this study, the effect of using thiourea (Tu) or Na_2_S as a sulfur source on the product structure was compared using hydrothermal synthesis at 190 °C for 7 days (ethylene glycol with water in the presence of poly(vinylpyrollidone) was used as a solvent). When Tu was used, CMTS precipitated in the form of concentric microspheres, 1–1.5 µm in size, consisting of hexagonal (in the cores) and tetragonal (the rims) forms. Most probably, the rapidly formed hexagonal nucleus was later surrounded by a slower-forming rim with a tetragonal structure. In contrast, when Na_2_S was used as a precursor, microspheres were not formed and a fine crystalline material with a homogeneous tetragonal structure was obtained. This allowed for the choice of micromorphology and product structure during synthesis.

## 1. Introduction

Photovoltaics are starting to play a significant role in supplying the growing demand for energy around the world. Therefore, the development of renewable energy production has become increasingly important over the past decade. Sustainability requires the development of solar cell manufacturing technologies built from materials that are harmless to the environment, non-toxic, and cost effective [1,2,3,4]. Meeting these energy needs will allow us to identify and address other important issues related to civilization, such as health, environmental safety, and sustainability.

Among the highly interesting materials that may find applications in photovoltaic technology are chalcogenides. These crystalline or amorphous compounds with covalent structures contain at least one of the following: S, Se, or Te (known as chalcogenide elements). Syntheses of semiconductor-based chalcogenides, such as Cu_2_XSnS_4_ (X = Fe, Co, Ni, Mn, or Zn), have inspired much research on these and other related materials, and experiments to improve their properties and behavior continue to be developed [5].

A chalcopyrite-based light-absorbing material based on Cu_2_ZnSnS_4_ (CZTS) is the best investigated among the various materials that have been developed. The theoretical calculations and experimental results indicated that it could have one of three types of crystal structures: kesterite, stannite, or wurtzite [6]. The difference between them lies in the different arrangements of Cu^+^ and Zn^2+^ in the crystal structure. The formation of the wurtzite-structured CZTS is not thermodynamically favorable, as indicated by the theoretical calculations, because the total energy of wurtzite-structured CZTS is much higher than that of kesterite and stannite [7]. This is responsible for the rare experimental observation of this crystal phase [8,9,10]. This is significant because the crystal structure of a light-absorbing material determines its optoelectronic properties, which, in turn, affect its performance in PV devices [8]. Considering the need for materials with multiple applications, researchers have developed various methods for chalcogenide synthesis and have attempted to discover the reasons for their crystallization in different structures [8,9,10,11,12].

Zou et al. [9] found that the nature of the sulfur precursor determines the formation and phase purity of the CZTS material synthesized by the hydrothermal reaction. Furthermore, this phase-control mechanism can be expanded to other quaternary Cu_2_MSnS_4_ (M = Cd^2+^, Mn^2+^) nanocrystal syntheses, where the relative reactivity of M^2+^ and the sulfur precursor plays a key role. Cu_2_MnSnS_4_ (CMTS) has several promising properties in common with Cu_2_ZnSnS_4_, which has been extensively studied for photovoltaic applications since it contains only non-toxic Earth-abundant elements and appropriate absorption properties for absorber materials [13].

CMTS is a semiconductor with multiple applications [14,15]. Thus far, solar cells based on Cu_2_MnSnS_4_ absorbers with conversion efficiencies of up to PCE = 1.8% have been developed [16], which form a tetragonal crystal structure similar to kesterite. It has a direct bandgap at 1.61 eV and a high absorption coefficient [17]. Therefore, it may be resistant to cation disorder, similar to the stannite Cu_2_FeSnS_4_. Liang et al. describe the synthesis of stannite and wurtzite CMTS nanocrystals via a hot injection approach [13].

The aim of our study was to investigate the effect of the nature of the sulfur precursor in the hydrothermal reaction on the formation, structure, and phase purity of the synthesized CMTS materials. Synthetic CMTS powders were prepared using a hydrothermal reaction at 190 °C for 7 days. Ethylene glycol with water in the presence of poly(vinylpyrollidone) (PVP) was used as a solvent. The effects of using Na_2_S or thiourea (Tu) as the sulfur source were compared. The structure and microstructure of synthetic CMTS depend, among other things, on the nature of these precursors. The development of methods to synthesize CMTS as an absorber in solar cells containing abundant metals that are commonly found on Earth is important to the photovoltaic community due to the similarity of its crystal structure and bandgaps compared with CZTS/CZTSSe, alongside its increased abundance of Mn compared to Zn.

## 2. Materials and Methods

### 2.1. Materials

Analytical-grade ethylene glycol (EG), copper (II) chloride dihydrate (CuCl_2_·2H_2_O), tin (IV) chloride pentahydrate (SnCl_4_·5H_2_O), manganese chloride tetrahydrate (MnCl_2_·4H_2_O), thiourea (Tu), sodium sulfide nonahydrate (Na_2_S·9H_2_O), poly(vinylpyrollidone) (PVP), and double-distilled water were used in the synthesis.

### 2.2. Synthesis of Microspheres

CMTS microparticles were synthesized using two different sulfur precursors: organic source sulfur (thiourea CH_4_N_2_S) and inorganic source sulfur (Na_2_S). Solutions of 1 mmol of copper (II) chloride dihydrate, 0.5 mmol of tin (IV) chloride pentahydrate, 0.5 mmol of ferrous chloride tetrahydrate, 0.5 mmol of manganese chloride tetrahydrate, and 2.5 mmol of thiourea (Tu) or sodium sulfide (Na_2_S) were mixed in proportions in which the molar ratio of Cu, Mn, Sn, and S was equal to 2:1:1:4. In these experiments, 0.512 g of CuCl_2_·2H_2_O, 0.297 g of MnCl_2_·4H_2_O, and 0.338 g of SnCl_2_·2H_2_O were dissolved in 12 mL of ultrapure water and 84 mL of ethylene glycol (EG). The solution was mixed with sulfur precursor (0.571 g of thiourea CH_4_N_2_S (Tu) or 1.801 g of Na_2_S·9H_2_O) and with 1.68 g of PVP. PVP surfactant is commonly used to reduce the size of nanoparticle agglomerates [18].

The mixture with approximately 96 mL of reagents was transferred to two Teflon-lined autoclaves and solvothermally treated at 190 °C for 7 days. The products were washed with water and acetone several times, centrifuged, and air-dried at 60 °C. In the remainder of the paper, samples synthesized using different methods are referred to according to the sulfur precursors used: CMTS_Tu—synthesized from thiourea, and CMTS_Na_2_S—synthesized from an inorganic sulfur source. In the first step of the formation mechanism of CMTS, metal chlorides, thiourea, and Na_2_S were dissolved in the solvent [19,20]:

CuCl_2_ → Cu^2+^ + 2Cl^−^ (1)

MnCl_2_ → Mn^2+^ + 2Cl^−^ (2)

SnCl_2_ → Sn^2+^ + 2Cl^−^ (3)

SC(NH_2_)_2_ → S^2−^ + H^+^ + NH_2_−C=NH or Na_2_S → 2Na^+^ + S^2−^ (4)

In the case of thiourea, a metal–thiourea complex was also formed in the solution:

Cu^2^^+^ + Mn^2^^+^ + Sn^4^^+^ + n(SC(NH)) → [CuMnSn(CS(NH))]^+^ (5)

The overall chemical reaction for the CMTS formation is given by:

2CuCl_2_ + MnCl_2_ + SnCl_2_ + 4CH_4_N_2_S + 8H_2_O → Cu_2_MnSnS_4_ + 8NH_4_Cl↑ + 4CO_2_↑ (6)

or:

2CuCl_2_ + MnCl_2_ + SnCl_2_ + 4Na_2_S → Cu_2_MnSnS_4_ + 8NaCl (7)

### 2.3. Characterization of Microspheres

The morphology of CFTS microspheres was characterized using scanning electron microscopy (SEM) with an FEI Versa 3D FEG and FEI Sirion 200 (Waltham, MA, USA) equipped with secondary electron (SE) and back-scattered electron (BSE) detectors. Samples of powders were fixed on a substrate with double-stick tape and imaged while uncoated in a low vacuum. Selected samples were immersed in epoxy and polished to reveal the internal structure of the particles. For metallographic studies, the specimens were ground with SiC grinding paper and successively polished with 6, 3, and 1 μm diamond abrasives. Detailed microstructure characteristics and transmission Kikuchi diffraction (TKD) analyses were performed on an FEI Versa 3D FEG-SEM scanning electron microscope (SEM; FEI, Hillsboro, OR, USA) equipped with a field emission gun (FEG) and an energy dispersive X-ray spectrometer (EDX; EDAX, Mahwah, NJ, USA). The details of the resulting microstructure of the particles, especially at the nanoscale, were revealed using a Tecnai TF 20 X-TWIN (200 kV) transmission electron microscope (TEM; TECNAI, Hillsboro, OR, USA) equipped with an energy-dispersive X-ray (EDS) spectrometer manufactured by EDAX (Mahwah, NJ, USA). For the TEM and TKD investigations, the lemella was prepared using the focused ion beam (FIB) lift-out technique in a Quanta 200i (FEI, Hillsboro, OR, USA) dual-beam FIB-SEM. TKD pattern simulation was realized using an Esprit DynamicS (Bruker Nano Gmbh, Berlin, Germany). The powder X-ray diffraction (XRD) patterns were recorded with a RigakuSmartLab diffractometer (Neu-Isenburg, Tokyo, Japan) in the range of 2θ = 2–75° with a step size of 0.05° using graphite-monochromatized Cu Kα radiation. The phases were identified using the ICCD database and XRAYAN software (v 4.0.5, “KOMA”—Henryk Marciniak, Warszawa, Poland) [21]. The refinements were performed using the General Structure Analysis System (GSAS) program created by Larson and Von Dreele [22]. For the Raman spectroscopy, a Thermo Scientific DXR Raman Microscope was used. The spectra were recorded at room temperature with a green laser (λ = 532 nm, laser power of 10 mW, slit aperture of 25 μm, and resolution of 1.9 cm^−1^ in the range between 100 and 3579 cm^−1^). A total of 10 exposures of 3 s each was taken for each spectrum. The interpretation of the spectra was performed with the aid of OMNIC for the Dispersive Raman software. The diffuse reflection spectrum was measured using a UV-Vis-NIR spectrometer (Shimadzu, UV-3150, Kyoto, Japan).

## 3. Results and Discussion

### X-ray Diffraction

**Figure 1:**
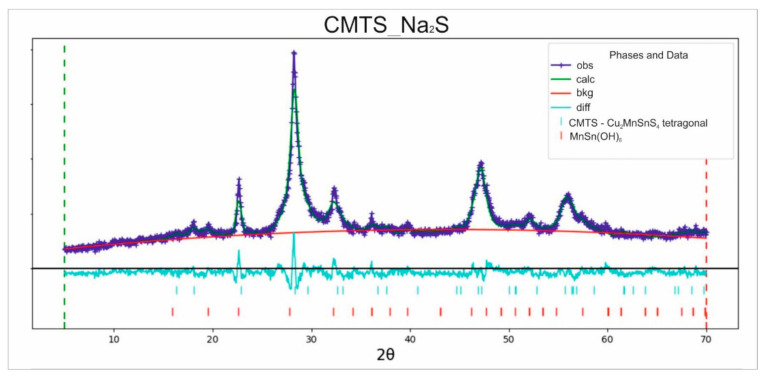
Refined XRD pattern of CMTS_Na_2_S consisting of tetragonal Cu_2_MnSnS_4_ with a small admixture of MnSn(OH)_6_ (data residual wR = 7.56%).

Figure 1 shows the XRD pattern of the microparticles synthesized with the use of Na_2_S as a precursor. It matched very well with the standard Cu_2_MnSnS_4_ pattern (standard JCPDS 51-0757) and corresponded with a tetragonal stannite structure. Three major peaks at 28.22°, 47.1°, and 55.9°, corresponding to (112), (220), and (312) lattice planes, respectively, were observed. The results were in good agreement with the previously reported data for the tetragonal structure of Cu_2_MnSnS_4_ [23]. Moreover, small amounts of MnSn(OH)_6_ (the synthetic equivalent of wicmannite) produced a diffraction peak around 2θ = 22°. This was formed by the precipitation of excess reactants. This contamination could be easily removed by washing with HCl [24].

**Figure 2:**
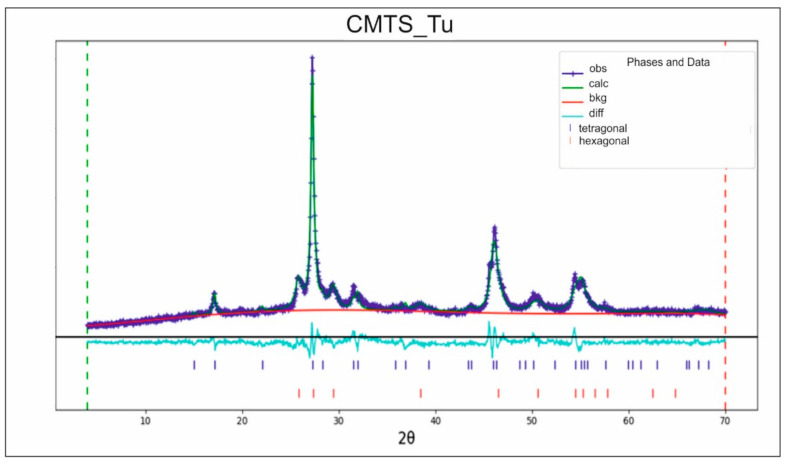
Refined XRD pattern of CMTS_Tu consisting of a mixture of tetragonal (62 wt.%) and hexagonal (38 wt.%) forms of Cu_2_MnSnS_4_ (data residual wR = 8.21%).

In contrast, the Cu_2_MnSnS_4_ synthesized from thiourea (Tu) as the sulfur precursor (CMTS_Tu) showed a very different XRD pattern (Figure 2). Besides the three XRD diffraction peaks of the tetragonal structure mentioned above, peaks at 26.7°, 30.3°, 39.5°, and 51.1° were also observed. These peaks matched the diffraction patterns of (100), (101), (102), and (103) planes of the hexagonal structure, respectively [23]. It is worth noting that, similar to the tetragonal structure, the hexagonal structure also had X-ray diffraction peaks at around 28.2°, 47.1°, and 56.4°. Therefore, these three peaks could be ascribed to either the (112), (220), and (312) planes of a tetragonal or the (002), (230), and (232) planes, respectively, of a hexagonal structure. Nevertheless, the weak diffraction peaks at around 18.1° and 32.5°, which were unique to the (101) and (200) planes, respectively, in tetragonal form, showed the existence of a tetragonal structure in CMTS_Tu (standard JCPDS 51-0757). This indicated that the synthesized CMTS_Tu material had a crystal structure in both the tetragonal phase and the hexagonal phase. Using Rietveld refinement, this sample was found to consist of a 62 wt.% tetragonal phase and a 38 wt.% hexagonal phase.

**Figure 3:**
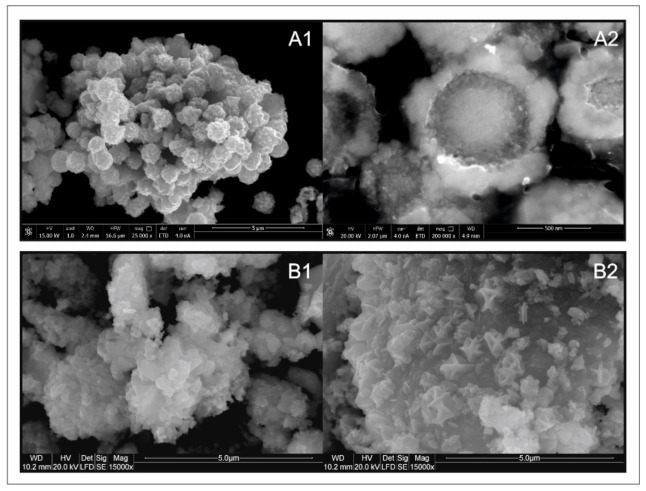
SEM images of (**A**) CMTS_Tu and (**B**) CMTS_Na_2_S. Images (**A1**,**B1**,**B2**) present the surfaces of powder samples. Image (**A2**) presents a polished sample to reveal the internal nanostructure of the microspheres on the cross-sections.

The morphologies and sizes of the Cu_2_MnSnS_4_ microparticles synthesized using different methods were characterized using scanning electron microscopy. The images of CMTS_Tu are shown in Figure 3A1,A2. The CMTS_Tu microparticles were spherical with sharp edges and had an average size of 1–1.5 µm (Figure 3A1). It is worth noting that all CMTS_Tu microparticle agglomerates (microspheres) had a generally monodisperse size distribution. The cross-sections showed that the microspheres had a concentric structure and consisted of two parts: a rim and a core (Figure 3A2). The external edge (rim) was composed of distinct small crystals, while the internal part (core) was much more homogeneous. In contrast, CMTS_Na_2_S crystallites had an irregular, blade-like, and sharp-edged shape and did not form spherical agglomerates (Figure 3B1,B2). The average diameter of the blades was close to 0.5–1 µm, and the size distribution was more polydisperse than that of CMTS_Tu.

**Figure 4:**
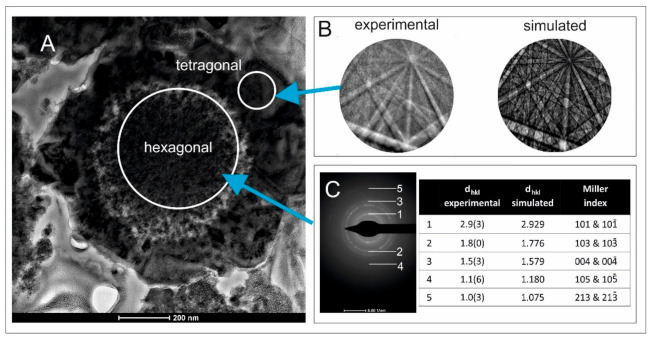
(**A**) TEM image of the concentric structure of a single CMTS_Tu microsphere. (**B**) Kikuchi diffraction patterns resulting from electron diffraction (EBSD) in the outer rim compared with those simulated for a relevant tetragonal structure. (**C**) Selected area diffraction pattern (TEM/SAED) resulting from electron diffraction in the inner core. A comparison between the measured and simulated d_hkl_ values listed in the table indicated the hexagonal structure of the core.

Figure 4A shows a TEM image of a single microsphere obtained via CMTS_Tu synthesis. Similar to the SEM images, a concentric structure consisting of a rim and a core was visible. The thickness of the rim was approximately 100–200 nm and the diameter of the core was equal to 600 nm. A TKD analysis was successfully performed on the outer rim. The image of the obtained Kikuchi lines was sharp and clear, indicating the good crystallinity of the material (Figure 4B). Each Kikuchi line was associated with Bragg diffraction from a single set of lattice planes and could be labeled using the same Miller indices that were used for identifying the diffraction spots. A high number of bands, consistent with a tetragonal structure, were apparent, which, compared to the simulated pattern, provided a clear indication that the outer rim was composed of a tetragonal phase. The selected area’s electron diffraction pattern (TEM/SAED) at the core of the microsphere is presented in Figure 4C. The presence of a set of distinct diffraction rings indicated the good crystallinity of the material. A comparison of the determined and simulated interplanar distances d_hkl_ for the lattice planes with Miller indices of (101), (103), (004), (105), and (213) indicated a hexagonal structure. The determination of the co-occurrence of CMTS with tetragonal and hexagonal structures in the CMTS_Tu synthesis product was consistent with the XRD analysis results presented above. Detailed crystal data and atomic coordinates for both tetragonal (zincblende type) and hexagonal (wurtzite type) structures of Cu_2_MnSnS_4_ are provided in [23].

**Figure 5:**
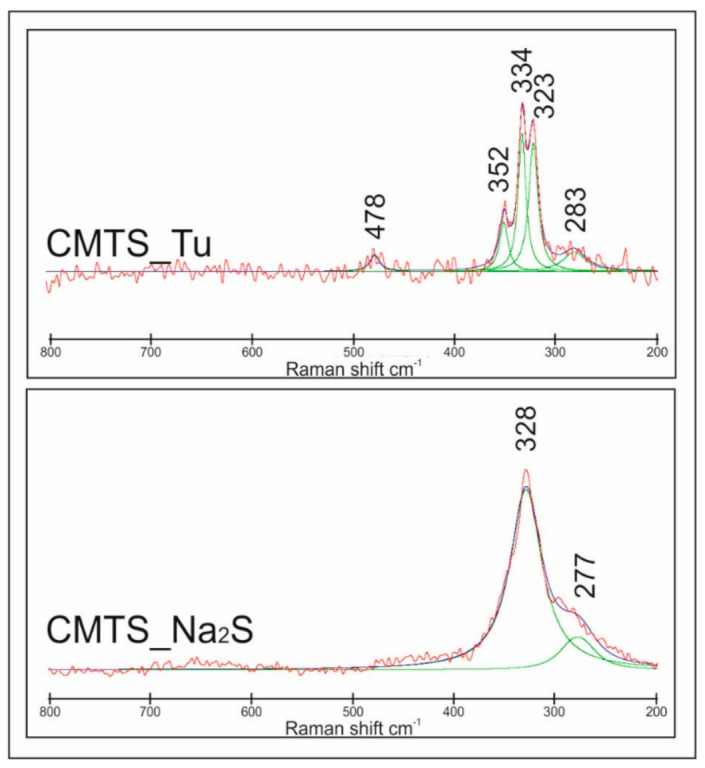
Raman spectra of CMTS_Tu and CMTS_Na_2_S. The band characteristic of the tetragonal structure shows the frequency variations. The main vibrational mode occurred at ~330 cm^−1^.

The Raman spectra for CMTS_Tu and CMTS_Na_2_S were similar (Figure 5). The dominant vibration at approximately ~330 cm^−1^, which was apparent in the experimental spectra, was that of the pure S anion mode around the solid Sn cations. The bands at approximately ~290 cm^−1^ could be regarded as the pure S anion mode around the Cu cation. The deconvolution of the CMTS_Tu spectrum to Lorentzian curves allowed us to notice an additional weaker band at 352 cm^−1^. This was consistent with the frequencies reported in the literature for this compound [25]. The splitting of the main peak at 323 and 334 cm^−1^ was present in the spectra. This effect was consistent with the XRD results. The main Raman peak of the CZTS stannite structure is typically reported as being within 330–339 cm^−1^, which is consistent with the values that were reported for films and crystals. Furthermore, frequencies in the range 320–334 cm^−1^ can also be found in the literature, a range in which the strongest Raman features were often reported for wurtzite and stannite CZTS [26].

**Figure 6:**
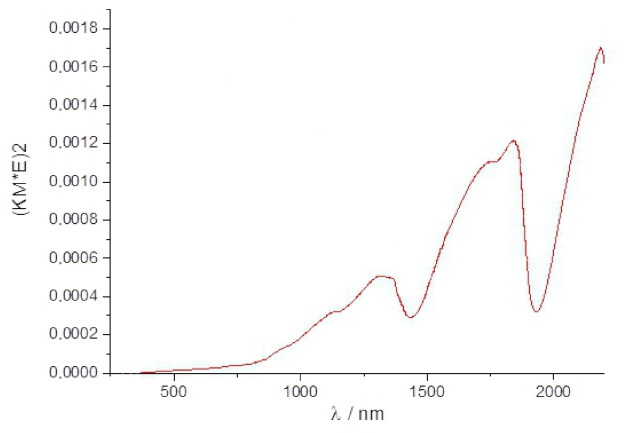
Reflectance UV-Vis spectrum for the CMTS_Tu sample.

The CMTS bandgap of 1.6 eV was first determined by Prabhakar et al. [27]. A diffuse reflection spectrum of the CMTS_Tu sample measured using a UV-VIS-NIR spectrometer is presented in Figure 6. Two strong and broad absorption peaks at ca. 1600–1900 nm and 1000–1400 nm are apparent. The strong absorption peaks extended to about 300–400 nm and consisted of smaller peaks. No steep absorption edge was observed that would allow for calculating the band gap and the measurement was inconclusive. This was probably due to the simultaneous presence of two forms of CMTS in the sample: hexagonal and tetragonal.

## 4. Conclusions

Two different sulfur sources were successfully used to synthesize Cu_2_MnSnS_4_ particles via the solvothermal method. The experiments showed that the type of sulfur precursor used played an important role in influencing the morphology and crystal structure of the CMTS particles. Changing the reactivity of the sulfur precursor allowed us to control the properties of the synthesized product. If the synthesis was performed using Tu as the sulfur source, CMTS precipitated as microspheres composed of a mixture of hexagonal forms (occurring in the microsphere core) and tetragonal forms (forming the microsphere rim). On the other hand, if Na_2_S was used as the precursor, microspheres were not formed and we obtained a fine crystalline material with a uniform tetragonal structure. The choice of the product’s micro-morphology influences the technology of its applications, e.g., for the production of films and thin-film layers. On the other hand, the choice of the crystal structure affects the total optoelectric properties.

The mutual relationship between hexagonal and tetragonal structures of CMTS was revealed for the first time. The presence of hexagonal forms in the nucleus of microspheres indicated their rapid formation during the initial stages of the synthesis. Subsequently, nanometer-sized spherical aggregates were surrounded by a slower-forming rim with a tetragonal structure. On the other hand, when Na_2_S was the only source of S, a tetragonal structure was formed. This was experimental confirmation of the effect predicted by [9]. The type of sulfur precursor influenced the structure of the resulting product through the different reactivity of the metal with sulfur: a faster reaction led to a hexagonal structure and a slower reaction led to a tetragonal structure. This synthesis method avoided the possible need for additional complicated and energy-consuming product treatment processes to enable structural changes, such as the proposed post-annealing step at 500 °C in sulfur vapor [28].

## Data Availability

The data can be requested from the corresponding author.

## References

[1] Le Donne A., Trifiletti V., Binetti S. (2019). New Earth-Abundant Thin Film Solar Cells Based on Chalcogenides. Front. Chem..

[2] Ramanujam J., Singh U.P. (2017). Copper indium gallium selenide based solar cells—A review. Energy Environ. Sci..

[3] Hou H., Guan H., Li L. (2017). Synthesis of Cu_2_FeSnS_4_ thin films with stannite and wurtzite structure directly on glass substrates via the solvothermal method. J. Mater. Sci. Mater. Electron..

[4] Sarilmaz A., Özel F., Karabulut A., Orak I., Şahinkaya M.A. (2020). The effects of temperature and frequency changes on the electrical characteristics of hot-injected Cu2MnSnS4 chalcogenide-based heterojunction. Phys. B Condens. Matter.

[5] Kocyigit A., Yıldırım M., Sarılmaz A., Ozel F. (2019). The Au/Cu2WSe4/p-Si photodiode: Electrical and morphological characterization. J. Alloy. Compd..

[6] Chen S., Gong X.G., Walsh A., Wei S.-H. (2009). Crystal and electronic band structure of Cu2ZnSnX4 (X=S and Se) photovoltaic absorbers: First-principles insights. Appl. Phys. Lett..

[7] Chen S., Walsh A., Luo Y., Yang J.-H., Gong X.G., Wei S.-H. (2011). Erratum: Wurtzite-derived polytypes of kesterite and stannite quaternary chalcogenide semiconductors [Phys. Rev. B82, 195203 (2010)]. Phys. Rev. B.

[8] Tiong V.T., Zhang Y., Bell J.M., Wang H. (2014). Phase-selective hydrothermal synthesis of Cu_2_ZnSnS_4_ nanocrystals: The effect of the sulphur precursor. CrystEngComm.

[9] Zou Y., Su X., Jiang J. (2013). Phase-Controlled Synthesis of Cu_2_ZnSnS_4_ Nanocrystals: The Role of Reactivity between Zn and S. J. Am. Chem. Soc..

[10] Li Z., Lui A.L.K., Lam K.H., Xi L., Lam Y.M. (2014). ChemInform Abstract: Phase-Selective Synthesis of Cu_2_ZnSnS_4_ Nanocrystals Using Different Sulfur Precursors. ChemInform.

[11] Lin Y.-H., Das S., Yang C.-Y., Sung J.-C., Lu C.-H. (2015). Phase-controlled synthesis of Cu_2_ZnSnS_4_ powders via the microwave-assisted solvothermal route. J. Alloys Compd..

[12] Long F., Chi S., He J., Wang J., Wu X., Mo S., Zou Z. (2015). Synthesis of hexagonal wurtzite Cu_2_ZnSnS_4_ prisms by an ultrasound-assisted microwave solvothermal method. J. Solid State Chem..

[13] Rudisch K., Espinosa-García W.F., Osorio-Guillén J.M., Araujo C.M., Platzer-Björkman C., Scragg J.J.S. (2019). Structural and Electronic Properties of Cu_2_MnSnS_4_ from Experiment and First-Principles Calculations. Phys. Status Solidi (b).

[14] Guan H., Wang X., Huang Y. (2018). Optical, photocatalytic and thermoelectric properties of Cu_2_MeSnS_4_ (Me = Mn^2+^, Fe^2+^, Co^2+^) nanocrystals via microwave-assisted solvothermal method. Chalcogenide Lett..

[15] Guan H., Hou H., Li M., Cui J. (2017). Photocatalytic and thermoelectric properties of Cu_2_MnSnS_4_ nanoparticles synthesized via solvothermal method. Mater. Lett..

[16] Li X., Hou Z., Gao S., Zeng Y., Ao J., Zhou Z., Da B., Liu W., Sun Y., Zhang Y. (2018). Efficient Optimization of the Performance of Mn^2+^ -Doped Kesterite Solar Cell: Machine Learning Aided Synthesis of High Efficient Cu_2_(Mn,Zn)Sn(S,Se)_4_ Solar Cells. Sol. RRL.

[17] Yu J., Deng H., Zhang Q., Tao J., Sun L., Yang P., Chu J. (2018). The role of sulfurization temperature on the morphological, structural and optical properties of electroplated Cu2MnSnS4 absorbers for photovoltaics. Mater. Lett..

[18] Cristóbal-García J.D., Paraguay-Delgado F., Herrera-Pérez G., Sato-Berrú R.Y., Mathews N.R. (2018). Polyvinylpyrrolidone influence on physical properties of Cu_2_ZnSnS_4_ nanoparticles. J. Mater. Sci. Mater. Electron..

[19] Maldar P., Gaikwad M., Mane A., Nikam S., Desai S., Giri S., Sarkar A., Moholkar A. (2017). Fabrication of Cu_2_CoSnS_4_ thin films by a facile spray pyrolysis for photovoltaic application. Sol. Energy.

[20] Dridi S., Aubry E., Bitri N., Chaabouni F., Briois P. (2020). Growth and Characterization of Cu_2_MnSnS_4_ Thin Films Synthesized by Spray Pyrolysis under Air Atmosphere. Coatings.

[21] Marciniak H., Diduszko R., Kozak M. (2006). XRAYAN-Program do rentgenowskiej analizyfazowej.

[22] Von Dreele R.B., Larson A.C. (1994). General Structure Analysis Sytem (GSAS).

[23] Liang X., Guo P., Wang G., Deng R., Pan D., Wei X. (2012). Dilute magnetic semiconductor Cu2MnSnS4 nanocrystals with a novel zincblende and wurtzite structure. RSC Adv..

[24] Rudashevskiy N.S. (1983). Tetrawickmanite, Tetragonal MnSn(OH)6—A New Mineral From North Carolina, And The Stottite Group. Int. Geol. Rev..

[25] Gürel T., Sevik C., Çağın T. (2011). Characterization of vibrational and mechanical properties of quaternary compounds Cu_2_ZnSnS_4_ and Cu_2_ZnSnSe_4_ in kesterite and stannite structures. Phys. Rev. B.

[26] Havryliuk Y., Valakh M.Y., Dzhagan V., Greshchuk O., Yukhymchuk V., Raevskaya A., Stroyuk O., Selyshchev O., Gaponik N., Zahn D.R.T. (2018). Raman characterization of Cu_2_ZnSnS_4_ nanocrystals: Phonon confinement effect and formation of CuxS phases. RSC Adv..

[27] Prabhakar R.R., Zhenghua S., Xin Z., Baikie T., Woei L.S., Shukla S., Batabyal S.K., Gunawan O., Wong L.H. (2016). Photovoltaic effect in earth abundant solution processed Cu_2_MnSnS_4_ and Cu_2_MnSn(S,Se)_4_ thin films. Sol. Energy Mater. Sol. Cells.

[28] Jiang H., Dai P., Feng Z., Fan W., Zhan J. (2012). Phase selective synthesis of metastable orthorhombic Cu_2_ZnSnS_4_. J. Mater. Chem..

